# Mutations in *BHD* and *TP53* genes, but not in *HNF1β* gene, in a large series of sporadic chromophobe renal cell carcinoma

**DOI:** 10.1038/sj.bjc.6603492

**Published:** 2006-11-28

**Authors:** S Gad, S H Lefèvre, S K Khoo, S Giraud, A Vieillefond, V Vasiliu, S Ferlicot, V Molinié, Y Denoux, N Thiounn, Y Chrétien, A Méjean, M Zerbib, G Benoît, J M Hervé, G Allègre, B Bressac-de Paillerets, B T Teh, S Richard

**Affiliations:** 1 Génétique Oncologique EPHE, CNRS FRE-2939, Institut de Cancérologie Gustave Roussy, 94800 Villejuif, France; 2 Faculté de Médecine Paris-Sud, 94270 Le Kremlin-Bicêtre, France; 3 Laboratory of Cancer Genetics, Van Andel Research Institute, Grand Rapids, MI 49503, USA; 4 Laboratoire de Génétique, Hôpital Herriot, 69003 Lyon, France; 5 Laboratoire d'Anatomie Pathologique, Hôpital Cochin, AP-HP, 75014 Paris, France; 6 Laboratoire d'Anatomie Pathologique, Hôpital Necker, AP-HP, 75015 Paris, France; 7 Laboratoire d'Anatomie Pathologique, Hôpital de Bicêtre, AP-HP, 94270 Le Kremlin-Bicêtre, France; 8 Laboratoire d'Anatomie Pathologique, Hôpital Foch, 92150 Suresnes, France; 9 Service d'Urologie, Hôpital Necker, AP-HP, 75015 Paris, France; 10 Service d'Urologie, Hôpital Cochin, AP-HP, 75014 Paris, France; 11 Consultation d'Oncogénétique Spécialisée, Service d'Urologie, Hôpital de Bicêtre, AP-HP, 94270 Le Kremlin-Bicêtre, France; 12 Service d'Urologie, Hôpital Foch, 92150 Suresnes, France; 13 Service de Génétique, Institut de Cancérologie Gustave Roussy, 94800 Villejuif, France

**Keywords:** chromophobe renal cell carcinoma, *BHD*, *TP53*, *HNF1β*, mutation, polymorphism

## Abstract

*BHD*, *TP53*, and *HNF1β* on chromosome 17 were studied in 92 cases of renal cell carcinoma (46 chromophobe, 19 clear cell, 18 oncocytoma, and nine papillary). Six, thirteen, and zero cases had, respectively *BHD*, *TP53*, and *HNF1β* mutations, (84% mutations involved chromophobe), suggesting a role for *BHD* and *TP53* in chromophobe subtype.

Renal cell carcinoma (RCC) is mainly comprised of clear cell, papillary, and chromophobe subtypes. The study of hereditary kidney cancer syndromes has led to the identification of kidney cancer-related genes that are also involved in sporadic RCC. Recently, germline *BHD* mutations were found in patients with Birt–Hogg–Dubé (BHD) syndrome (Nickerson *et al*, 2002), and a diverse spectrum of renal tumours have been described with somatic inactivation of *BHD* reported in BHD-related renal tumours (Khoo *et al*, 2002; Schmidt *et al*, 2005; Vocke *et al*, 2005). *BHD* promoter methylation has been reported in a subset of sporadic clear cell and chromophobe RCC, but somatic mutation of *BHD* in sporadic cases is rare (da Silva *et al*, 2003; Khoo *et al*, 2003). *BHD* is located at 17p11.2, and LOH has been detected in all RCC subtypes (Khoo *et al*, 2003; Vocke *et al*, 2005). The *TP53* gene is located at 17p13.1 near *BHD*. A study has reported 30% of somatic missense mutations of *TP53* in chromophobe with LOH of chromosome 17p, suggesting that *TP53* plays a role in this subtype (Contractor *et al*, 1997). The *HNF1β* gene (hepatocyte nuclear factor), which was found mutated in patients with maturity-onset diabetes of the young (MODY5), is located at 17q12 (Horikawa *et al*, 1997). Biallelic inactivation of this gene has been reported in two of 12 patients with chromophobe RCC; both patients have germline *HNF1β* mutations, and their tumours showed LOH, suggesting inactivation through the classic two-hit hypothesis (Rebouissou *et al*, 2005).

Multiple losses of whole chromosomes were frequently found in chromophobe RCC, especially in chromosome 17 (Speicher *et al*, 1994). We hypothesised that the lost chromosomal regions may harbour chromophobe RCC-specific tumour suppressor genes, and their inactivation contributes to the tumorigenesis. Here, we focused on three cancer-related genes located at chromosome 17, *BHD*, *TP53*, and *HNF1β*, and examined their involvement in chromophobe RCC by studying 46 cases and compared with 19 clear cell, 18 oncocytoma, and nine papillary subtypes. We screened these tumours for mutations, evaluated the *BHD* promoter methylation status, and estimated the allelic frequencies of polymorphisms in these genes.

## MATERIALS AND METHODS

### Tissue samples and DNA extraction

Ninety-two frozen sporadic renal tumour samples were collected from various hospitals in France and USA (French Kidney Tumour Consortium and Cooperative Human Tissue Network). All patients are of Caucasian origin. This included two patients with bilateral chromophobe RCC but without evidence of genetic predisposition. This study was performed after approval from our local Ethics Committee. Informed consent was obtained from each patient. Genomic DNA was extracted using the QIAamp DNA Mini Kit (Qiagen, Courtaboeuf, France) according to the manufacturer's instructions.

### Sequencing analysis

The entire coding region of *BHD*, *TP53*, and *HNF1β* was screened for mutations by direct sequencing (Nickerson *et al*, 2002; B. Bressac-de Paillerets, unpublished data; Rebouissou *et al*, 2005; see Supplementary Information).

### SNP analysis

Intronic and exonic SNPs (iSNPs and eSNPs) were obtained from the sequencing results. Rare homozygous genotypes have the lowest allelic frequencies according to the Hardy–Weinberg's law. We used the allelic frequencies of HapMap–CEU (http://www.hapmap.org).

### Methylation analysis of the *BHD* promoter region

Genomic DNA were incubated with or without *Hpa*II (Invitrogen, Cergy-Pontoise, France). Polymerase chain reaction (PCR) was then performed with specific primers (available on request) and products were analysed on standard agarose gels. The presence of methylated cytosines was determined by comparing the same sample under the digested or nondigested conditions, that is, if cytosines were methylated, *Hpa*II would not be cleaved at the restriction enzyme sites, and PCR amplification would be successful.

### Statistical analysis

*χ*^2^ test was used to compare the mutation frequencies as well as the frequencies of rare homozygous genotypes of each polymorphism in each tumour subtype. When the conditions of application of *χ*^2^ could not be obtained, a Yates's correction was applied, or the Fisher's exact test was used. Statistical significance was indicated by *P*<0.05.

## RESULTS AND DISCUSSION

### Alterations in *BHD*

**Figure 1:**
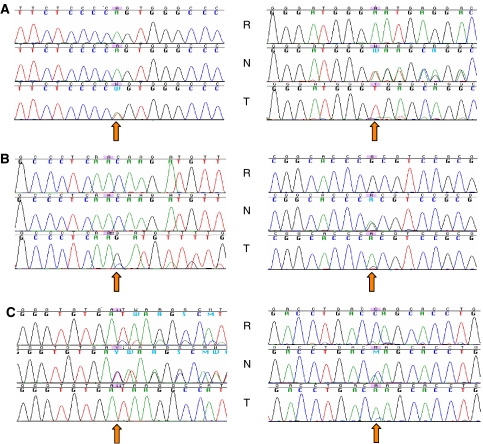
Sequence chromatograms for *BHD*, *TP53*, and *HNF1β*. R, N, and T are DNA from a commercially available reference, the normal tissue and its matched tumour tissue, respectively. (**A**) Corresponds to *BHD* with a somatic mutation (T68, c.1433(IVS12)-2A>T) (left) and a possible germline mutation (T16, c.103_125(558_580)del23) (right). (**B**) Corresponds to *TP53* with a somatic mutation (T72, c.393_395delCAA) (left) and a possible germline mutation (T9, c.467G>A) (right). (**C**) Corresponds to *HNF1β* with a cytosine insertion in intron 8 (left) and a SNP in the non-coding region of exon 9 (c.^*^99C>) (right).

Two nonsense, three frameshift, and three predicted splice mutations were identified in six samples, five of 46 chromophobe RCC (10.9%) and one of 18 oncocytomas (5.6%) (Table 1). This is the first report of somatic *BHD* mutations in sporadic chromophobe RCC and renal oncocytoma. T16 and T35 exhibited their respective mutations in both tumour and corresponding matched normal tissue, showing possible germline mutations, although contamination of tumour cells in the normal tissue could not be ruled out completely. Unfortunately, the blood DNA of these patients could not be obtained to verify their germline mutation status. T16 also showed loss of the wild-type allele but retained mutant strand in its tumour tissue (LOH) (Figure 1A). Two chromophobe RCC (T68 and T87b) showed a double mutation in each of the tumours. In patient A (T87a and T87b), we detected two novel somatic mutations and a previously described germline alteration (Schmidt *et al*, 2005), which was confirmed with the patient's blood DNA. Although patient A did not show any evidence of genetic predisposition, he has bilateral chromophobe RCC, and a germline mutation makes him a potential hereditary case and was referred to genetic counsellors. T68 showed two distinct somatic mutations not found in the matched normal tissues. The second somatic mutation is a possible second hit, instead of LOH, further supporting the tumour suppressive role of *BHD*. We did not detect any mutation at the hot spot within exon 11 as reported in BHD patients. The *BHD* mutation frequency in chromophobe is statistically not significant compared to the other subtypes (*P*>0.20, with Yates's correction). Methylation status of the *BHD* promoter was analysed on 61 of 92 samples (39 chromophobe, seven clear cell, and 15 oncocytoma), which had sufficient DNA quantity to perform the enzymatic digestion. No evidence of *BHD* promoter methylation was found.

### Mutations in *TP53*

Eight missense, three frameshift, one in-frame, and one predicted splice mutations were identified in 13 tumours, 11 of 46 chromophobe (23.9%), one of 19 clear cell (5.3%), and one of nine papillary RCC (11.1%). The mutation in T75 is located at the last base of exon 4 that can induce a splicing effect (Holmila *et al*, 2003). A known hot spot mutation in sarcomatoid RCC (Oda *et al*, 1995) has been detected in one papillary (T26) and one chromophobe (T63). A sarcomatoid component can occur in all subtypes, and its presence indicates poor outcome (Cheville *et al*, 2004). The matched normal tissue of T9, a clear cell subtype, has the same mutation as its tumour tissue, suggesting a possible germline mutation (Figure 1B). However, no blood DNA was available to confirm its germline status. All *TP53* mutations detected here have been described in the *TP53* database (www-p53.iarc.fr/P53aim.html). The *TP53* mutation frequency in chromophobe is statistically significant compared to the other subtypes (*P*<0.01). Therefore, *TP53* mutations occur preferentially in chromophobe as reported (Contractor *et al*, 1997). The high percentage of *TP53* mutations in chromophobe could reflect the different pathways in its tumorigenesis, compared to other subtypes.

### Analysis of *HNF1β*

No mutations were identified in all coding exons of the *HNF1β* gene. However, an insertion of a cytosine in the intron 8 was detected (Table 2, and Table 3 in Supplementary Information). This is a deletion/insertion polymorphism (DIP) that has been reported previously (Horikawa *et al*, 1997). Here the frequency of its rare genotype (insC) in chromophobe is statistically significant compared to the other subtypes (*P*<0.02). Furthermore, this variant was observed in a normal tissue sample, but was lost in the matched tumour through LOH (Figure 1C). We could not establish any relationship between *HNF1β* and sporadic RCC as we did not find any mutations, suggesting *HNF1β* mutation as a very rare genetic event in sporadic renal tumours.

### Analysis of SNPs in *BHD*, *TP53*, and *HNF1β*

We detected 14 SNPs, including one possible new iSNP in *BHD* (Table 2 and Supplementary Table 3). All tumours that carry the *BHD* mutations showed homozygosity in all four iSNPs. All except two *TP53-*mutated tumours (T26 and T45) demonstrated homozygous alleles in all four SNPs. In addition, the proportion of samples showing homozygous SNP alleles in *BHD*, *TP53*, and *HNF1β* are 41/92 (44.6%), 65/92 (70.6%), and 37/92 (40.2%), respectively. Chromophobe RCC exhibited the highest percentage of rare homozygous genotypes (Table 2). Among the 92 renal tumours, 24 samples are homozygous for all SNP studied (26%). Twenty-one of them are of chromophobe subtype (45.6% of all chromophobe; *P*<0.001). One of them carries a *BHD* mutation and eight of them have *TP53* mutations (72.7% of *TP53*-mutated chromophobe; *P*<0.02). The other three are of oncocytoma subtype (16.7% of all oncocytoma). Homozygous SNP alleles detected in this study may indicate chromosomal deletions. In samples with mutations, it may suggest LOH, which is consistent with the two-hit hypothesis. However, we also noticed the high frequency of homozygous SNP alleles especially in samples without *TP53* mutation (70%). Although it has been shown that p53 is functional in p53 wild-type RCC cells (Warburton *et al*, 2005), the relationship between chromosomal 17 deletions and *TP53*, especially in sporadic chromophobe subtype, is worth further investigation.

In summary, *BHD* and *TP53* may play an important role as tumour suppressors in chromophobe RCC.

## Figures and Tables

**Table 1:** Description of mutations detected in *BHD* and *TP53* genes in 92 sporadic renal tumours

**Gene**	**Sample**	**Cell type**	**Exon**	**Mutation description**	**Mutation type**	**Mutated protein**	**Mutation origin**
*BHD*	T16	Chromophobe RCC	4	c.103_125(558_580)del23	Frameshift	p.Asn35fs	Possible germline
	T35	Chromophobe RCC	9	c.919(1374)G>T^a^	Nonsense	p.Glu307X	Possible germline
	T87a	Chromophobe RCC	9	c.995_998(1450_1453)del4	Frameshift	p.Leu332fs	Somatic
	T87b	Chromophobe RCC	9	c.1062(IVS9)+2T>G	Predicted splice mutation	x	Germline
	T87b	Chromophobe RCC	11	c.1179(1634)delC	Frameshift	p.Thr393fs	Somatic
	T68	Chromophobe RCC	11	c.1177(IVS10)-6delCCT	Predicted splice mutation	x	Somatic
	T68	Chromophobe RCC	13	c.1433(IVS12)-2A>T	Predicted splice mutation	x	Somatic
	T55	Oncocytoma	14	c.1659(2114)G>A	Nonsense	p.Trp553X	Somatic
							
*TP53*	T70	Chromophobe RCC	4	c.150_157del8^b^	Frameshift	p.Ile50fs	ND
	T75	Chromophobe RCC	4	c.375G>A^c^	Predicted splice mutation	p.Thr125Thr	ND
	T72	Chromophobe RCC	5	c.393_395delCAA	In frame deletion	p.Asn131del	Somatic
	T41	Chromophobe RCC	5	c.469G>T	Missense	p.Val157Phe	Somatic
	T66	Chromophobe RCC	6	c.569delC	Frameshift	p.Pro190fs	Somatic
	T34	Chromophobe RCC	6	c.644G>T	Missense	p.Ser215Ile	Somatic
	T33	Chromophobe RCC	7	c.757A>G	Missense	p.Thr253Ala	Somatic
	T43	Chromophobe RCC	8	c.817C>T	Missense	p.Arg273Cys	Somatic
	T63	Chromophobe RCC	8	c.832C>A	Missense	p.Pro278Thr	ND
	T45	Chromophobe RCC	8	c.877dupA	Frameshift	p.Gly293fs	ND
	T62	Chromophobe RCC	10	c.1009C>T	Missense	p.Arg337Cys	ND
	T9	Clear cell RCC	5	c.467G>A	Missense	p.Arg156His	Possible germline
	T26	Papillary RCC	8	c.832C>A	Missense	p.Pro278Thr	Somatic

Abbreviations: BHD=Birt–Hogg–Dubé; ND=Not determined owing to the unavailability of matched normal tissue.

a For *BHD,* c. corresponds to coding sequence relative to ATG in exon 4 (Genbank accession number NM_144997). Numbers in brackets are refering to the previous nomenclature used (Genbank accession number AF517523).

b For *TP53*, c. corresponds to coding sequence according to ATG in exon 2 (Genbank accession number NM_000546).

c Not a silent mutation because it involves the last base of exon 4 and has been reported to be responsible for exon skipping.

**Table 2:** Polymorphisms detected in *BHD,* *TP53*, and *HNF1β* genes in 92 sporadic renal tumours

				**Proportion of rare homozygous genotypes (%)^c^**				
**Gene**	**SNP ID^a^**	**Location**	**Description**	**All tumours *N*=92**	**Chromophobe RCC *N*=46**	**Clear-cell RCC *N*=19**	**Oncocytoma *N*=18**	**Papillary RCC *N*=9**
*BHD*	rs1736219	Intron 5	c.397-14C>T	28.3	17.4	4.3	4.3	2.2
	rs3744124	Intron 8	c.871+36G>A	0	0	0	0	0
	rs8065832	Intron 9	c.1062+6C>T	29.3	19.6	4.3	3.3	2.2
		Intron 12	c.1433-38A>G	15.2	12	2.2	1.1	0
								
*TP53*	rs1642785	Intron 2	c.74+38G>C	16.3	10.9	2.2	2.2	1.1
	rs1800370	exon 4	c.108G>A, p.P36P	1.1	1.1	0	0	0
	rs1042522	exon 4	c.215G>C, p.R72P	14.1	10.9	2.2	1.1	0
	rs1800372	exon 6	c.639A>G, p.R213R	1.1	1.1	0	0	0
								
*HNF1β*	rs2107133	Intron 6	c.1339+27T>C	4.3	4.3	0	0	0
		Intron 8	c.1653+47_48insC	7.6	7.6	0	0	0
	rs3110641	Intron 8	c.1654-22C>T	15.2	12	0	2.2	1.1
	rs8068014	exon 9	c.^*^47T>G^b^	1.1	0	0	0	1.1
	rs2229295	exon 9	c.^*^99C>A^b^	10.9	7.6	0	2.2	1.1
	rs1800929	exon 9	c.^*^100A>G^b^	2.2	0	0	1.1	1.1
	rs2689	exon 9	c.^*^274A>T^b^	27.2	17.4	3.3	5.4	1.1

Abbreviations: BHD=Birt–Hogg–Dubé; HNF=hepatocyte nuclear factor; RCC=renal cell carcinoma.

a SNP information was obtained from www.ncbi.nlm.nih.gov/SNP/.

b These 4 SNPs are located in exon 9 of *HNF1β* after the translation stop codon (Genbank accession number NM_000458).

c Rare homozygous genotypes are defined as genotypes having the lowest allelic frequency q^2^ according to the Hardy–Weinberg law and genotypes given in Table 3 (Supplementary Information on line).
