# Supplementary material for: Mutations in BHD and TP53 genes, but not in HNF1β gene, in a large series of sporadic chromophobe renal cell carcinoma
Source: Br J Cancer. 2006 Nov 28;96(2):336–40. doi: 10.1038/sj.bjc.6603492 (PMC2360004; doi:10.1038/sj.bjc.6603492)
Supplement: Supplementary Information [file 6603492x1.pdf]

***BHD* and *TP53* mutations in chromophobe RCC by Gad *et al.* BJC 2006**

SUPPLEMENTARY INFORMATION ON LINE

MATERIALS & METHODS : Sequencing analysis

The entire coding region (exons 4-14) of the *BHD* gene (also known as *FLCN* or folliculin gene, OMIM #607273, Genbank accession number NM_144997) was screened for mutations by direct sequencing with primer sequences and PCR conditions according to Nickerson *et al.* (Nickerson *et al,* 2002). The entire coding region (exons 2-10) of the *TP53* gene, coding the p53 protein (OMIM #191170, Genbank accession number NM_000546) was studied with primers and PCR conditions available upon request (B. Bressac-de Paillerets, unpublished data). For the *HNF1β* gene (Hepatocyte Nuclear Factor), also known as *TCF2* (OMIM #189907, Genbank accession number NM_000458), the entire coding region (exons 1-9) was analyzed according to primers and PCR conditions from Rebouissou *et al.* (Rebouissou *et al,* 2005). PCR products were analyzed on standard 1.5% agarose gels stained with ethidium bromide (0.5 μg/mL) before purification with ExoSAP-IT (Amersham Biosciences, Saclay, France). Sequencing reactions were performed using Big Dye Terminator (Applied Biosystems, Courtaboeuf, France), purified through Sephadex G-50 (Amersham Biosciences) and run on an ABI 3730 Genetic Analyzer (Applied Biosystems). We aligned and analyzed the sequences by Seqscape v2.1 (Applied Biosystems) or Sequencher v4.2.2 (Gene Codes Corporation, Ann Arbor, USA) softwares. All sequence alterations were verified by reamplifying the corresponding fragment and repeating the sequencing procedure using both forward and reverse primers. Matched normal DNA were also sequenced whenever available. The *BHD* alterations described in this paper were designated according to the recommendations of the recent nomenclature system for human gene mutations (Human Genome Variation Society: www.hgvs.org) with the coding sequence beginning at the start codon ATG and not at the non-coding exons (455 bp upstream) as reported in other previously published articles.
